# Comparison of the Effect of Fimasartan versus Valsartan on Blood Pressure Variability in Acute Ischemic Stroke: A Double-Blind Randomized Trial

**DOI:** 10.1155/2019/7836527

**Published:** 2019-06-02

**Authors:** Dong Hoon Shin, Soohwa Song, Yeong Bae Lee

**Affiliations:** ^1^Department of Neurology, Gil Medical Center, Gachon University College of Medicine, Incheon, Republic of Korea; ^2^Department of Biomedical Engineering, Gachon University of Medicine and Science, Incheon, Republic of Korea

## Abstract

Higher blood pressure variability (BPV) is associated with poor functional outcome and mortality in acute stroke. This randomized controlled trial was conducted to compare the effect on BPV between fimasartan and valsartan (Boryung Pharmaceutical Co., Ltd., Seoul, Republic of Korea) in patients with acute ischemic stroke. Eighty patients were randomly assigned to receive either valsartan or fimasartan after 7 days of acute ischemic stroke onset, for duration of 8 weeks. Of them, 62 patients completed the study [valsartan (n=31), fimasartan (n=31)]. We measured BP for 24 hours using ambulatory BP monitoring device before and after 8 weeks of starting BP medication. We calculated several indexes such as standard deviation (SD), weighted 24-hour BP with SD (wSD), coefficient of variation (CV), and average real variability (ARV) to assess BPV and to compare indexes of BPV between 2 drugs. SD values of systolic BP in daytime, nighttime, and 24 h period (15.55±4.02 versus 20.55±8.77,* P*=0.006; 11.98±5.52 versus 16.47±6.94,* P*=0.007; 17.22±5.30 versus 21.45±8.51,* P*=0.024), wSD of systolic BP (8.27±3.01 versus 10.77±4.18,* P*=0.010), and ARV of systolic BP (15.85±6.17 versus 19.68±7.83,* P*=0.040) of patients receiving fimasartan after 8 weeks were significantly lower than patients receiving valsartan. In paired* t*-test, SD values of daytime, nighttime, and 24 h period of systolic BP of patients receiving fimasartan were significantly decreased after 8 weeks (15.55±4.02 versus 18.70±7.04,* P*=0.038; 11.98±5.52 versus 17.19±7.35,* P*=0.006; 17.22±5.30 versus 20.59±5.91,* P*=0.015). Our study showed that fimasartan had greater effect on reducing BPV after acute ischemic stroke than valsartan. Trials registry number is KCT0003254.

## 1. Introduction

It is well known that higher mean blood pressure (BP) is associated with a high risk of all-cause mortality and cardiovascular morbidity and mortality [1]. The BP level continually fluctuates and BP (BPV) can be shown as beat-to-beat variability, 24-hour variability, day-to-day variability, and visit-to-visit variability. Moreover, BPV is another risk factor for cardiovascular events and is associated with target organ damage and all-cause mortality independent of the BP level [2–4]. While beat-to-beat variability or 24-hour variability reflects an increase in central sympathetic activity, a decrease in arterial or cardiopulmonary reflex, and an increase in arterial stiffness [5, 6], day-to-day variability or visit-to-visit variability is associated with increased arterial stiffness, improper dosing or titration of antihypertensive medication, or poor medication compliance [7–9].

An increase of 24-hour or daytime BPV is associated with an increased development of cardiac and vascular damage as well as with a greater incidence of cardiovascular morbidity and mortality irrespective of mean BP levels [10–13]. The importance not only of reducing mean BP but also of reducing BPV is recognized as a target to prevent cardiovascular events, especially stroke [9, 14]. In a recent systematic review, systolic BPV was reduced by calcium-channel blockers and nonloop diuretic drugs and increased by angiotensin-converting enzyme inhibitors, angiotensin-receptor blockers (ARB), and ß blockers [15]. However, recent studies reported that ARB with a long-term half-life could be superior in reducing BPV compared to ARB with a shorter half-life such as losartan [16–18]. Fimasartan is an ARB that has a potent and safe BP-lowering effect and has one of the longest half-lives among ARB [19, 20]. Therefore, we hypothesized that fimasartan would be more beneficial for reducing BPV in patients with acute ischemic stroke after 8 weeks of treatment compared with valsartan.

## 2. Subjects and Methods

### 2.1. Study Population

The* fimasartan on blood pressure variability in acute stroke (FIRST)* was an 8-week, prospective, single center, double-blind study. This study was approved by Gachon University Gil Medical Center Institutional Review Board (GAIRB2015-61). All participants provided written informed consent before enrollment. We prospectively enrolled 80 patients with acute ischemic stroke. The patients were registered in Gachon University Gil Medical Center from July 2015 to February 2018. Inclusion criteria consisted of (1) patients who had acute ischemic stroke with confirmation by diffusion-weighted image within 7 days of symptom onset (patients of transient ischemic attack were not included in the study) and (2) those who had a history of hypertension or should be treated with antihypertensive medication at discharge. Patients with the following conditions were excluded: secondary hypertension, congestive heart failure, severe valvular heart disease, malignant arrhythmia, renal insufficiency (serum creatinine concentration ≥ 2 mg/dL), severe liver disease, chronic inflammatory disease, and malignant disease (lifetime prognosis < 6 months). Clinical trial registration numbers and date of registration is KCT0003254 (11/Oct/2018).

### 2.2. Study Design

Eligible subjects were randomly allocated in a 1:1 ratio to receive fimasartan 60 mg or valsartan 80 mg using a computer generated block card randomization procedure by principle investigator and dose titration was permitted up to double the dose if BP-lowering was insufficient. Treatment was continued for 8 weeks. Demographic features and risk factors were recorded, including hypertension (defined as receiving medication for hypertension or blood pressure (BP) > 140/90 mm Hg on repeated measurements), diabetes mellitus (defined as receiving medication for diabetes mellitus, fasting blood sugar ≥ 126 mg/dL, or 2-hour postprandial blood sugar ≥ 200 mg/dL), hyperlipidemia (defined as receiving cholesterol-reducing agents or overnight fasting cholesterol level > 200 mg/dL), current (or no longer smoked < 6 months) cigarette smoking, history of stroke, and history of coronary heart disease. We performed the 24 h ambulatory BP monitoring (ABPM) before study medication and at 8 weeks after study medication. The ABPM was performed with an oscillometric-based device (TM-2431; A&D Co., Tokyo, Japan). Blood pressure recordings were made every 30 minutes during the daytime period (between 10:00 and 22:00) and every 60 minutes during the nighttime period (between 00:00 and 06:00). The pressure cuff was always placed on the nondominant arm. The ABPM was always started between 09:00 and 10:00. During the days the recordings were being taken, each subject was asked to fill in a diary card recording the times of going bed, getting up, taking medications, and any symptoms and events that may influence the BP. The mean value and standard deviation (SD) of the ambulatory BP of each subject were calculated for the entire 24 h period and separately for the daytime period and the nighttime period. The other parameters were calculated as follows: weighted 24-hour BP SD (wSD; computed as the average of day and night SDs, weighted for their respective durations), coefficient of variation (CV; SD divided by the mean), and average real variability (ARV; the average of absolute changes between consecutive BP readings). All methods were performed in accordance with Good Clinical Practice guidelines.

### 2.3. Primary and Secondary Outcomes

The primary outcome was difference of mean and SD of the entire 24 h period and separately for the daytime period and the nighttime period between two groups after treatment, which is measured by ABPM. Secondary outcomes were difference of other parameters for BPV such as wSD, CV, and ARV. Primary and secondary outcomes were evaluated at baseline and 8 weeks. All adverse events were reported by the investigators and adjudicated by an independent adjudication committee, being classified as serious or nonserious. Serious adverse events were defined as cardiovascular events or events requiring hospitalization.

### 2.4. Statistical Analysis

A sample size of 80 subjects, 40 in each arm, is sufficient to detect a clinically important difference of 4 between groups in reducing 24-hour systolic BP SD assuming a standard deviation of 5.5 using a two-tailed t-test of difference between means with 80% power and a 5% level of significance [21]. Considering a dropout rate of 33% the sample size required is 80 (40 per group). Continuous variables are presented as mean ± SD and categorical variables are presented as absolute value and proportion (%). The 2 groups of patients were compared using the independent* t*-test or chi-square test, as appropriate for continuous, and categorical variables, which included age, sex, hypertension, diabetes mellitus, cardiac problems, previous stroke, hypercholesterolemia, current cigarette smoking, and levels of hemoglobin, glucose, total cholesterol, C-reactive protein, homocysteine, and uric acid; in addition, mean BP, SD of BP, and other parameters for BPV were collected. Variables for BP and BPV at baseline and 8 weeks were compared using independent* t*-test between groups. Variables for BP and BPV at 8 weeks were compared with baseline using a paired* t*-test in each group. The software SPSS 19.0 (SPSS Inc., Chicago, Illinois, USA) was used for statistical analysis.

## 3. Results

### 3.1. Characteristics of the Study Participants

During the entry period, 80 patients were enrolled in this study. Of them, 62 patients (mean age, 58.3 years, and 48 male) completed the study (18 patients were lost to the study by withdrawal of consent (4), absence of follow-up (1), additional calcium-channel blocker (3), and pain from BP cuff compression (10)). The risk factors were as follows: hypertension in 28 patients (45.2%), diabetes in 12 patients (19.4%), atrial fibrillation in 1 patient (1.6%), cigarette smoking in 28 patients (45.2 %), and hyperlipidemia in 32 patients (51.6%). Three (4.8%) and 2 (3.2%) patients had histories of stroke and coronary heart disease, respectively.  Table S1 summarizes their baseline demographics. There was no significant difference in risk factors and baseline laboratory findings between two groups.

### 3.2. Comparison of BP-Lowering Effect: Fimasartan versus Valsartan

Table S2 shows that daytime, nighttime, and a 24 h period of systolic and diastolic BP were significantly reduced in patients receiving fimasartan (SBP; 133.0±22.2 versus 156.1±21.5,* P*<0.001; 122.6±17.9 versus 144.5±20.5,* P*<0.001; 129.5±19.1 versus 152.0±19.6,* P*<0.001; DBP; 80.1±13.1 versus 90.6±13.5,* P*=0.002; 74.4±10.0 versus 85.3±11.3,* P*<0.001; 78.1±9.8 versus 88.6±11.9,* P*<0.001, respectively) and valsartan (SBP; 146.4±18.7 versus 162.7±20.9,* P*<0.001; 131.1±19.9 versus 154.6±20.7,* P*<0.001; 140.6±17.2 versus 159.9±19.9,* P*<0.001; DBP; 86.0±11.9 versus 93.6±14.9,* P*=0.007; 78.1±11.9 versus 89.7±13.5,* P*<0.001; 83.2±10.9 versus 92.4±13.3,* P*<0.001, respectively) after 8 weeks in a paired test. Also, daytime and nighttime pulse pressure were significantly decreased in patients receiving fimasartan (52.9±16.3 versus 65.5±14.5,* P*<0.001; 48.2±12.3 versus 59.2±15.2,* P*=0.001) and valsartan (60.4±12.9 versus 69.1±14.3,* P*=0.002; 52.9±12.7 versus 65.0±12.4,* P*<0.001) after 8 weeks in a paired test. Compared with patients receiving valsartan in Table S3, patients receiving fimasartan of daytime and a 24 h period of systolic BP after 8 weeks were significantly reduced (146.4 ±18.7 versus 133.1±22.2,* P*=0.014; 140.6±17.2 versus 129.5±19.1,* P*=0.022, respectively). Figures S1 and S2 showed the summary of these results for BP-lowering effect of two drugs.

### 3.3. Comparison of Effects on 24-h ABPM BPV: Fimasartan versus Valsartan

**Figure 1:**
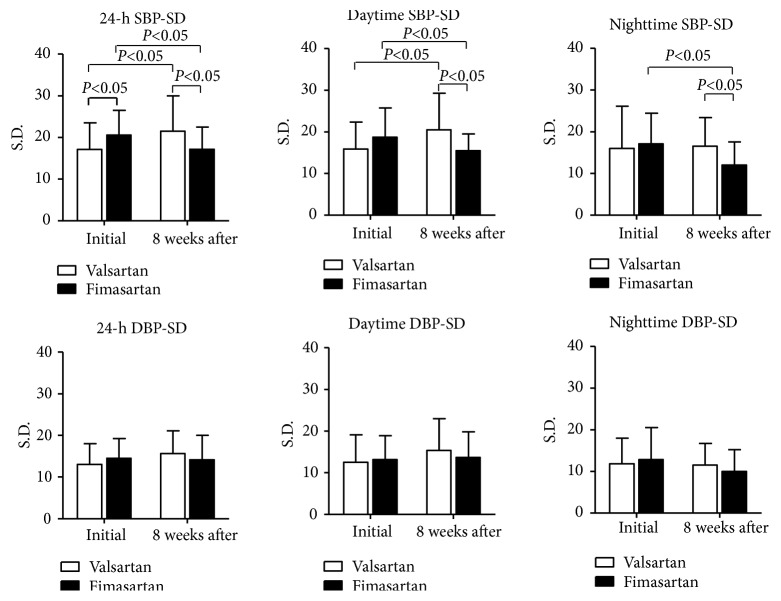
In paired test, while valsartan increased significantly SD of 24-hour, daytime, and nighttime systolic blood pressure (BP) from baseline, fimasartan reduced significantly SD of 24-hour, daytime, and nighttime of systolic BP from baseline. In comparison with valsartan, fimasartan reduced significantly SD of 24 hours, daytime, and nighttime of systolic BP after 8 weeks.

**Figure 2:**
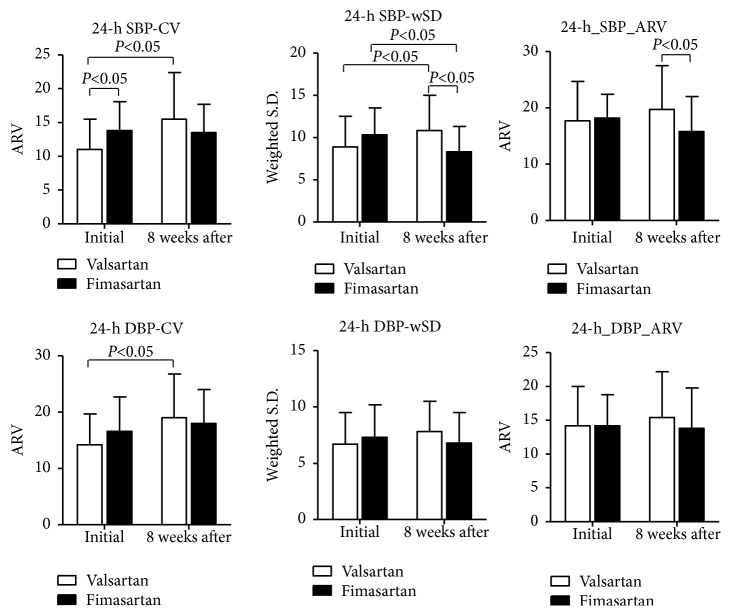
In paired test, while valsartan increased significantly 24-hour CV of systolic and diastolic blood pressure (BP) and 24-hour wSD of systolic BP from the baseline after 8 weeks, fimasartan reduced significantly 24-hour wSD of systolic BP from the baseline after 8 weeks. Comparing with valsartan, fimasartan reduced significantly 24-hour wSD and ARV of systolic BP after 8 weeks.

In comparison with valsartan (Tables 1 and 2), the SD of systolic BP in daytime, nighttime, and a 24 h period (15.55±4.02 versus 20.55±8.77,* P*=0.006; 11.98±5.52 versus 16.47±6.94,* P*=0.007; 17.22±5.30 versus 21.45±8.51,* P*=0.024, respectively), wSD of systolic BP (8.27±3.01 versus 10.77±4.18,* P*=0.010), and ARV of systolic BP (15.85±6.17 versus 19.68±7.83,* P*=0.040) of patients receiving fimasartan after 8 weeks were significantly lower than patients receiving valsartan. In paired* t*-test (Tables 3 and 4), the SD of daytime, nighttime, and a 24-hour period of systolic BP of patients receiving fimasartan were significantly decreased (15.55±4.02 versus 18.70±7.04,* P*=0.038; 11.98±5.52 versus 17.19±7.35,* P*=0.006; 17.22±5.30 versus 20.59±5.91,* P*=0.015) after 8 weeks. However, in patients receiving valsartan, there were significantly increased values of SD in the daytime and a 24-hour period of systolic BP (20.55±8.77 versus 15.90±6.47,* P*=0.020; 21.45±8.51 versus 17.14±6.38,* P*=0.027, respectively) and wSD and CV of systolic BP (10.77±4.18 versus 8.88±3.55,* P*=0.038; 15.51±6.91 versus 10.95±4.50,* P*=0.003) and 24 h SD and CV of diastolic BP (15.56±5.51vs. 13.03±5.00,* P*=0.050; 19.05±7.82 versus 14.24±5.48,* P*=0.004) after 8 weeks. Figures 1 and 2 showed the summary of these results for two drugs effect on BPV.

### 3.4. Adverse-Events Profile

The percentage of patients who experienced a nonserious adverse event or serious adverse event was similar in two groups (Table S4). Adverse events leading to drug discontinuation occurred in the valsartan (1 intracranial haemorrhage) and fimasartan (1 stroke recurrence) groups. The common causes of nonserious events were gastrointestinal problems, insomnia, constipation, headache, and dizziness.

## 4. Discussion

In the FIRST study, although both valsartan and fimasartan significantly reduced systolic and diastolic BP from baseline BP, fimasartan had a significantly greater reduction on daytime and the 24 h period of systolic BP than valsartan after 8 weeks for patients with acute ischemic stroke. Also, fimasartan significantly reduced BPV compared with baseline and compared with patients receiving valsartan, while valsartan aggravated BPV compared with baseline after it was administered for 8 weeks to patients with acute ischemic stroke.

In our study, fimasartan reduced daytime and the 24 h period of systolic BP significantly more than valsartan in patients with acute ischemic stroke. Hypertension is a well-established risk factor for the development of cardiovascular disease and randomized clinical trials in hypertensive patients clearly show that effective antihypertensive therapy for BP control prevent primary stroke occurrence [22]. Evidence from hypertension-treatment trials has shown that systolic BP reduction was linearly related to the lower risk of recurrent stroke, myocardial infarct, and any cardiovascular death. In addition, high BP during follow-up was associated with an increased risk of recurrent stroke [23, 24]. Therefore, fimasartan can be a more effective antihypertensive agent than valsartan for patients with acute ischemic stroke for preventing recurrent stroke.

In our study, 8 weeks of fimasartan treatment significantly reduced the SD of daytime, nighttime, and a 24-h period of systolic BP and 24-hour wSD of systolic BP from baseline. In addition, when compared with valsartan, fimasartan significantly improved the SD of daytime, nighttime, and the 24-h period of systolic BP, wSD, and ARV of systolic BP in acute ischemic stroke after 8 weeks. A recent meta-analysis showed that short-term BPV from ABPM including 24-h systolic BP SD, wSD, and 24-h ARV of systolic BP were significantly correlated with target organ damage like left ventricular mass index[25], and 24-hour BPV assessed by ARV is significantly associated with the presence and progression of subclinical organ damage and the incidence of cardiovascular events [26]. Fimasartan can reduce the short-term BPV, assessed by various indexes, in patients with acute ischemic stroke from baseline BPV and compared with valsartan after 8 weeks of treatment. This treatment may be beneficial for patients with acute ischemic stroke and high BPV.

BP measurement manifests continuous fluctuations of BP, and BPV can be classified as very short-term BPV (beat-by-beat), short-term BPV (within 24 h), and long-term BPV (day-by-day, visit-to-visit) [27]. Very short-term BPV and short-term BPV reflect increased central sympathetic drive, reduced arterial or cardiopulmonary reflex, and humoral and rheological factors. However, long-term BPV is associated with increased arterial stiffness, improper dosing or titration of antihypertensive medication, and poor medication compliance. Increased short-term and long-term BPV are associated with target organ damage such as cardiac, vascular, and renal damage and an increased incidence of cardiovascular events and mortality independent of mean BP level [2, 4, 14, 28]. In an acute stroke setting, greater BPV early after the acute stroke is associated with an increased risk of death and disability, greater lesion growth shown by diffusion-weighted imaging, worse clinical course, and the risk of intracranial haemorrhage in patients undergoing thrombolytic therapy [29–31].

The ARB have been shown to exert additional beneficial cardiovascular effects such as regression of cardiac hypertrophy, enhancement of endothelium-dependent relaxation, improvement of endothelial function and arterial stiffness, and antiatherosclerotic properties independent of BP reduction [32–36]. Because the variable effects of different class in antihypertensive agents on stroke risk reduction cannot be explained by effects on mean BP reduction alone, BPV can be a potential therapeutic target, and antihypertensive agents should be targeted toward stabilizing BPV in addition to controlling mean BP. Many classes of drugs are currently used to treat hypertension as a monotherapy or in combination, for example, diuretics, ß blockers, angiotensin-receptor blockers (ARB), renin inhibitors, angiotensin-converting enzyme inhibitors, and calcium-channel blockers.

In a recent systematic review, BPV determined by SD of 24-hour ABPM could be reduced by calcium-channel blockers and nonloop diuretics and could be increased by angiotensin-converting enzyme inhibitors, angiotensin-receptor blockers, and ß blockers. Valsartan did not significantly reduce BPV in patients with hypertension after 12 months of treatment [37] and valsartan increased the individual SD of morning systolic BP after it was additionally given to patients insufficiently controlled by amlodipine monotherapy in another study [18]. In the X-CELLENT study, candesartan did not reduce BPV evaluated by 24-hour BP monitoring after 3 months of treatment [38]. Telmisartan did not affect BPV of morning systolic BP after it was given to patients on amlodipine monotherapy [18], and it reduced BPV by suppression of sympathetic activity and improvement of the baroreceptor reflex in an animal study [39]. Based on result of this study, fimasartan is one of the ARB that can reduce BPV, although the mechanism of BPV reduction was not investigated.

This study has several limitations. Although the sample size was calculated considering the two-sample parallel design, the number of patients is relatively small. However, ABPM can reduce sample size requirements for clinical trials of hypertension [40] and could compensate for the weakness of the small population number of this study. Because the degree of short-term BPV is partially proportional to BP levels and patients treated with valsartan did not reach optimal target levels at last visit after 8 weeks, higher BPV in valsartan group could be due to suboptimal BP control. However, because we dosed up to double dosage in both groups when BP was above 140/90 mmHg at follow-up visit after 4 weeks, higher BPV in valsartan group can be due to the insufficient efficacy of valsartan. Although there was no significant difference in baseline BP in both groups, the difference on baseline BP might affect the result of this study because average BPs of valsartan group is higher than those of fimasartan group. Our results should be interpreted with caution because this is a single center study. Large prospective studies are needed to confirm our results.

To the best of our knowledge, the present study provides the first prospective evidence that fimasartan can improve short-term BPV measured by ABPM after acute ischemic stroke although the classification of fimasartan is ARB. The effect of fimasartan on reducing daytime and 24 h systolic BP and reducing short-term BPV more than valsartan could have positive effects on secondary stroke prevention shown by recent systematic data analysis, which showed that controlling BP highly during follow-up is associated with higher recurrence of stroke and high BPV is associated with poor functional outcome and mortality. Therefore, we can deduce that treatment with fimasartan for patients with acute ischemic stroke and with high short-term BPV could be beneficial.

## Figures and Tables

**Table 1:** BPV-SD.

	Initial			8 weeks after		
S.D.	Valsartan	Fimasartan	*P*-value	Valsartan	Fimasartan	*P*-value
Daytime SBP	15.90±6.47	18.70±7.04	.115	20.55±8.77	15.55±4.02	*.006*
Daytime DBP	12.51±6.59	13.24±5.66	.650	15.41±7.57	13.71±6.09	.343
Nighttime SBP	15.96±10.10	17.19±7.35	.593	16.47±6.94	11.98±5.52	*.007*
Nighttime DBP	11.78±6.20	12.87±7.64	.544	11.54±5.20	9.96±5.17	.243
24-h SBP	17.14±6.38	20.59±5.91	*.034*	21.45±8.51	17.22±5.30	*.024*
24-h DBP	13.03±5.00	14.45±4.76	.263	15.56±5.51	14.18±5.75	.346

Values are presented as the mean ± SD. *P* values were calculated using the independent t-test.

**Table 2:** BPV-the other parameters.

	Initial			8 weeks after		
S.D.	Valsartan	Fimasartan	*P*-value	Valsartan	Fimasartan	*P*-value
24-h SBP wSD	8.88±3.55	10.26±3.19	.118	10.77±4.18	8.27±3.01	*.010*
24-h DBP wSD	6.74±2.82	7.29±2.91	.457	7.84±2.71	6.82±2.72	.148
24-h SBP CV	10.95±4.50	13.76±4.25	*.016*	15.51±6.91	13.47±4.21	.173
24-h DBP CV	14.24±5.48	16.58±6.09	.121	19.05±7.82	18.02±5.98	.572
24-h SBP ARV	17.77±7.01	18.23±4.20	.756	19.68±7.83	15.85±6.17	*.040*
24-h DBP ARV	14.20±5.79	14.15±4.60	.968	15.44±6.82	13.82±6.00	.332

Values are presented as the mean ± SD. *P* values were calculated using the independent t-test.

**Table 3:** BPV-SD.

	Valsartan			Fimasartan		
S.D.	Initial	8 weeks	*P*-value	Initial	8 weeks	*P*-value
Daytime SBP	15.90±6.47	20.55±8.77	*.020*	18.70±7.04	15.55±4.02	*.038*
Daytime DBP	12.51±6.59	15.41±7.57	.122	13.24±5.66	13.71±6.09	.751
Nighttime SBP	15.96±10.10	16.47±6.94	.803	17.19±7.35	11.98±5.52	*.006*
Nighttime DBP	11.78±6.20	11.54±5.20	.861	12.87±7.64	9.96±5.17	.116
24-h SBP	17.14±6.38	21.45±8.51	*.027*	20.59±5.91	17.22±5.30	*.015*
24-h DBP	13.03±5.00	15.56±5.51	.050	14.45±4.76	14.18±5.75	.834

Values are presented as the mean ± SD. *P* values were calculated using the paired t-test.

**Table 4:** BPV-the other parameters.

	Valsartan			Fimasartan		
S.D.	Initial	8 weeks	*P*-value	Initial	8 weeks	*P*-value
24-h SBP wSD	8.88±3.55	10.77±4.18	*.038*	10.26±3.19	8.27±3.01	*.013*
24-h DBP wSD	6.74±2.82	7.84±2.71	.066	7.29±2.91	6.82±2.72	.518
24-h SBP CV	10.95±4.50	15.51±6.91	*.003*	13.76±4.25	13.47±4.21	.769
24-h DBP CV	14.24±5.48	19.05±7.82	*.004*	16.58±6.09	18.02±5.98	.352
24-h SBP ARV	17.77±7.01	19.68±7.83	.244	18.23±4.20	15.85±6.17	.101
24-h DBP ARV	14.20±5.79	15.44±6.82	.326	14.15±4.60	13.82±6.00	.790

Values are presented as the mean ± SD. *P* values were calculated using the paired t-test.

## Data Availability

The data used to support the findings of this study are available from the corresponding author upon request.

## Supplementary Materials

Supplementary Materials Table S1. Demographics of patients enrolled in FIRST study. Table S2. BP-Averages. Table S3. BP-Averages. Table S4. Adverse Event Profiles during 8-week treatment. Figure S1. These figures showed the comparison of trends of 24-hour blood pressure of initial and after 8 weeks in fimasartan group and valsartan group. Figure S2. Both of fimasartan and valsartan reduced significantly 24 hours, daytime, and nighttime of systolic blood pressure (BP) from the baseline after 8 weeks medication. Comparing with valsartan group, fimasartan group showed greater drop of 24-hour and daytime of systolic BP at 8 weeks.
