# Supplementary material for: Comparison of the Effect of Fimasartan versus Valsartan on Blood Pressure Variability in Acute Ischemic Stroke: A Double-Blind Randomized Trial
Source: Cardiovasc Ther. 2019 Jun 2;2019:7836527. doi: 10.1155/2019/7836527 (PMC6739756; doi:10.1155/2019/7836527)

Table S1. Demographics of patients enrolled in FIRST study.

	Valsartan (n=31)	Fimasartan (n=31)	*P*
Age, years	57.5±11.5	59.1±12.6	*0.593*
Male, n (%)	24 (77.4)	24 (77.4)	*1.000*
Risk factors, n (%)			
Hypertension	13 (41.9)	15 (48.4)	0.610
Diabetes mellitus	3 (9.7)	9 (29.0)	0.054
Atrial fibrillation	0 (0)	0 (0)	1.000
Hyperlipidemia	16 (51.6)	16 (51.6)	1.000
Cardiac disease	1 (3.2)	1 (3.2)	1.000
Previous stroke	1 (3.2)	2 (6.5)	0.554
Smoking	12(40)	16(51.6)	0.363
Baseline laboratory findings			
Hemoglobin, g/dl	14.9±1.7	14.7±1.5	0.661
Glucose, mg/dl	128.3±38.9	149.4±61.4	0.112
Total cholesterol, mg/dl	199.7±33.1	201.3±42.5	0.868
CRP, mg/dl	0.27±0.37	0.30±0.38	0.773
Homocysteine, mg/dl	14.1±3.6	15.1±6.9	0.446
Uric acid, mg/dl	15.7±5.0	18.0±5.9	0.624
Stroke subtype, n (%)			0.701
LAD	9 (29.0)	10 (32.3)	
SAD	16 (51.6)	18 (58.1)	
CE	3 (9.7)	2 (6.5)	
*Others	3 (9.7)	1 (3.2)	
Baseline NIHSS, median (IQR)	3 (1-5)	3 (1-5)	0.815
Double dosing, n (%)	2 (6.5)	1 (3.2)	
Average dose at study end, mg	85.2±20.0	62.0±10.8	
Average interval from start to double dosing, day	35.5	31	
Average number of measurement of ABPM, n	24.8	24.5	0.664

CE indicates cardioembolism; LAD, large artery disease; NIHSS, NIH stroke scale; SAD, small artery disease; *others were comprised of cryptogenic embolism and hematologic coagulopathy.

Table S2. BP-Averages

mmHg	Valsartan			Fimasartan		
	Initial	8 weeks	*P*-value	Initial	8 weeks	*P*-value
Daytime SBP	162.7±20.9	146.4±18.7	**<.001**	156.1±21.5	133.0±22.2	**<.001**
Daytime DBP	93.6±14.9	86.0±11.9	**.007**	90.6±13.5	80.1±13.1	**.002**
Nighttime SBP	154.6±20.7	131.1±19.9	**<.001**	144.5±20.5	122.6±17.9	**<.001**
Nighttime DBP	89.7±13.5	78.1±11.9	**<.001**	85.3±11.3	74.4±10.0	**<.001**
24-h SBP	159.9±19.9	140.6±17.2	**<.001**	152.0±19.6	129.5±19.1	**<.001**
24-h DBP	92.4±13.3	83.2±10.9	**<.001**	88.6±11.9	78.1±9.8	**<.001**

Values are presented as the mean ± SD. *P*-values were calculated using the paired t-test (mean ± SD).

Table S3. BP-Averages

mmHg	Initial			8 weeks after		
	Valsartan	Fimasartan	*P*-value	Valsartan	Fimasartan	*P*-value
Daytime SBP	162.7±20.9	156.1±21.5	.234	146.4±18.7	133.0±22.2	**.014**
Daytime DBP	93.6±14.9	90.6±13.5	.424	86.0±11.9	80.1±13.1	.076
Nighttime SBP	154.6±20.7	144.5±20.5	.063	131.1±19.9	122.6±17.9	.088
Nighttime DBP	89.7±13.5	85.3±11.3	.183	78.1±11.9	74.4±10.0	.188
24-h SBP	159.9±19.9	152.0±19.6	.131	140.6±17.2	129.5±19.1	**.022**
24-h DBP	92.4±13.3	88.6±11.9	.250	83.2±10.9	78.1±9.8	.059

Values are presented as the mean ± SD. *P*-values were calculated using the independent t-test.

Table S4. Adverse Event Profiles during 8-week treatment

Adverse events	Valsartan group (n=31)	Fimasartan group (n=31)	*P*
**Serious adverse events**	1 (3.2%)	1 (3.2%)	1.000
Fracture	0	0	
Intracranial haemorrhage	0	0	
Retinitis	1	0	
Stroke recurrence	0	1	
**Non-serious adverse events**	18 (58.1%)	19 (61.3%)	0.796
Bradycardia	3	1	
GI problem	2	2	
Constipation	0	1	
Anxiety	3	1	
Headache	0	3	
Itching	1	1	
Insomnia	1	0	
Joint pain	0	2	
Common cold	0	1	
Dizziness	1	1	
Urinary frequency	2	0	
Weight loss	1	1	
Liver enzyme elevation	0	2	
Tremor	1	0	
Hyperglycaemia	0	0	
Hiccup	0	0	
Ear problem	0	0	
Epistaxis	0	0	
Confusion	0	0	
Hernia	0	0	
Haematuria	1	0	
Anorexia	0	0	
Pulmonary infection	1	0	
Erectile dysfunction	1	0	
Tachycardia	0	1	
Dyspnea	0	1	
Paresthesia	0	1	

GI=gastrointestinal

Supplementary Figure legends

Figure S1. These figures showed the comparison of trends of 24-hour blood pressure of initial and after 8 weeks in fimasartan group and valsaratan group.

Figure S2. Both of fimasartan and valsartan reduced significantly 24-hour, daytime, and nighttime of systolic blood pressure (BP) from the baseline after 8 weeks medication. Comparing with valsartan group, fimasartan group showed greater drop of 24-hour and daytime of systolic BP at 8 weeks.

Supplementary figure 1

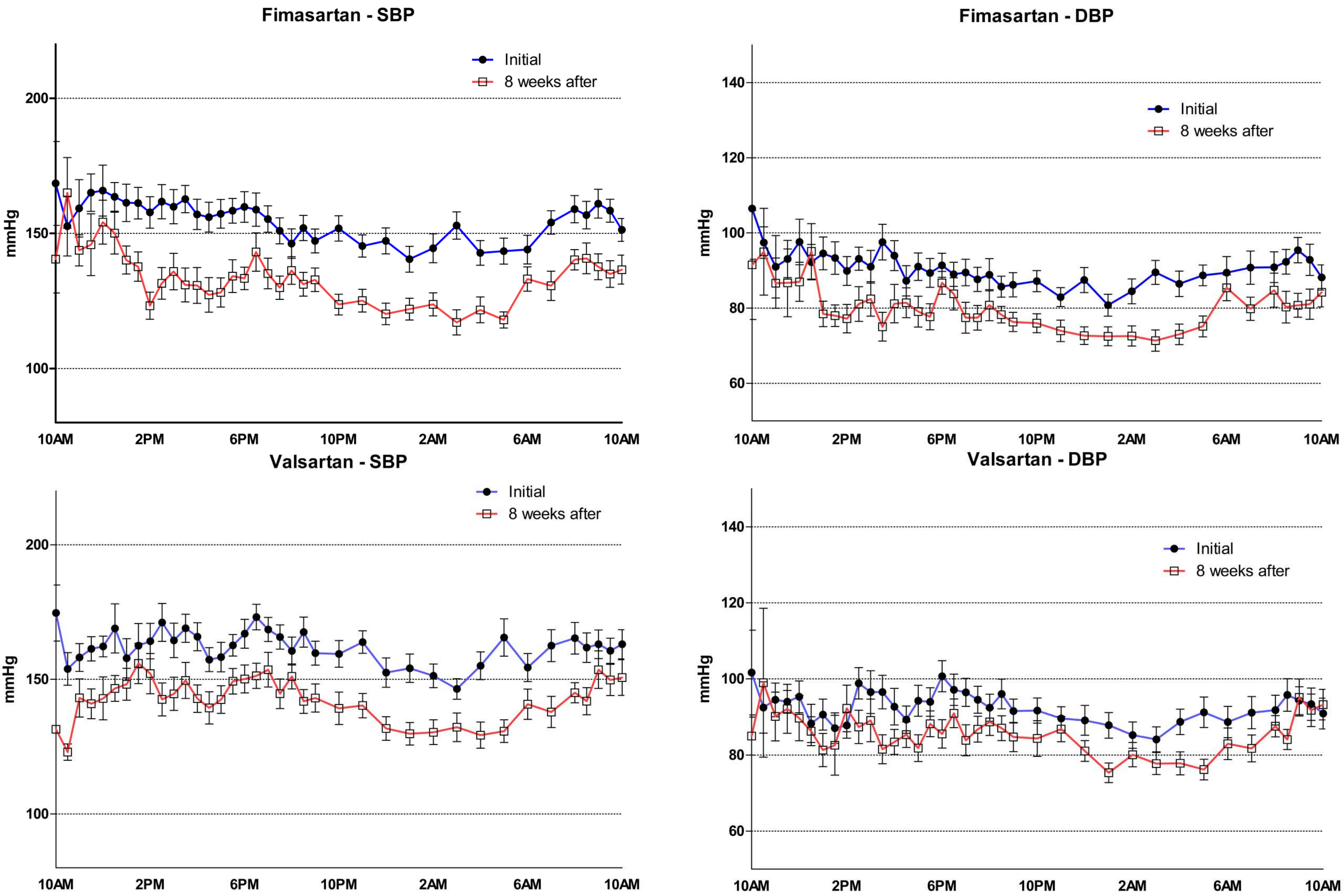

Supplementary figure 2

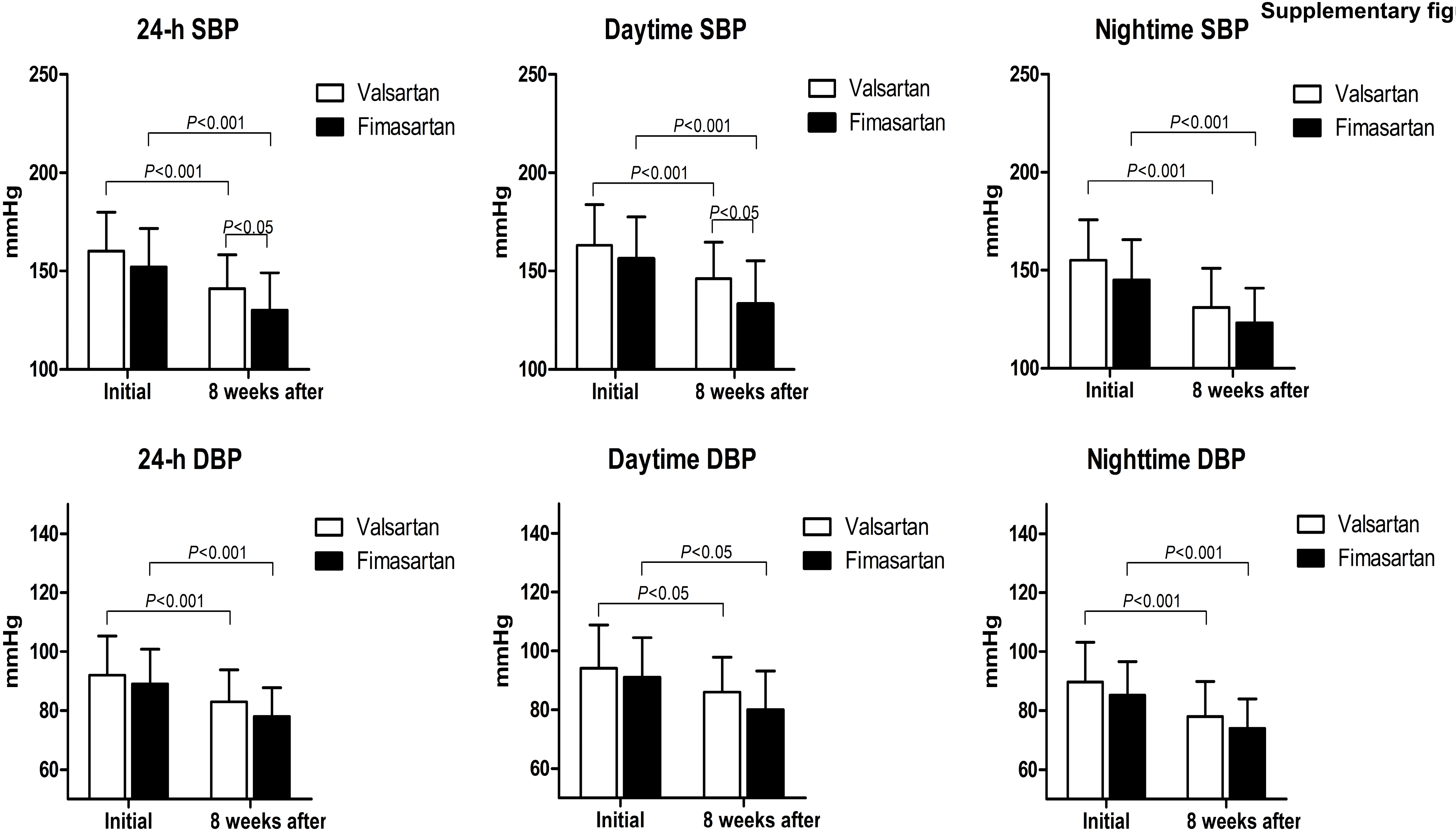

Supplement: Supplementary Materials — Table S1. Demographics of patients enrolled in FIRST study. Table S2. BP-Averages. Table S3. BP-Averages. Table S4. Adverse Event Profiles during 8-week treatment. Figure S1. These figures showed the comparison of trends of 24-hour blood pressure of initial and after 8 weeks in fimasartan group and valsartan group. Figure S2. Both of fimasartan and valsartan reduced significantly 24 hours, daytime, and nighttime of systolic blood pressure (BP) from the baseline after 8 weeks medication. Comparing with valsartan group, fimasartan group showed greater drop of 24-hour and daytime of systolic BP at 8 weeks. [file 7836527.f1.pdf]
